# Expansion of a Web-Based Platform to Support Caregivers in California Using CareNav

**DOI:** 10.1093/geroni/igab046.1672

**Published:** 2021-12-17

**Authors:** Heather Young, Janice Bell, Kathleen Kelly, Tina Kilaberia, Jennifer Mongoven

**Affiliations:** 1 University of California Davis, Sacramento, California, United States; 2 University of California, Davis, Sacramento, California, United States; 3 Family Caregiver Alliance, San Francisco, California, United States

## Abstract

About one in five Americans is engaged in providing care to a family member. Caregivers (unpaid family members or friends) support older adults and persons with disability with a variety of conditions, including challenges in physical, cognitive, and mental health. In California, 4.5 million family caregivers are assisting individuals over the age of 18. The CA Department of Health Care Services funds 11 Caregiver Resource Centers (CRC) to support caregivers and, in 2019, provided support to expand information technology services through adoption of a statewide online assessment platform and client portal, CareNav™, to serve as a client record and referral tool. CareNav™ facilitates collection of consistent state-wide data that can inform program improvement and policy. This study evaluated the implementation process from the perspective of 35 CRC team members in guided focus group discussions. CRC staff identified several potential benefits to adopting CareNav™, including ease of client access and convenience, the ability to aggregate data to inform planning and policy, and a streamlined process for resource sharing. Challenges included customizing site-specific data needs, as well as concerns about equitable access to internet services. Ongoing evaluation will focus on validation and visualization of data, and translation of data into actionable information to improve quality and reach.

